# The absence of myelin basic protein promotes neuroinflammation and reduces amyloid β-protein accumulation in Tg-5xFAD mice

**DOI:** 10.1186/1742-2094-10-134

**Published:** 2013-11-05

**Authors:** Ming-Hsuan Ou-Yang, William E Van Nostrand

**Affiliations:** 1 Departments of Neurosurgery & Medicine, Stony Brook University, Stony Brook, NY 11794-8122, USA

**Keywords:** Alzheimer’s disease, Amyloid β-protein, Astrocyte, Chaperone molecules, Matrix metalloproteinases, Microglia, Myelin basic protein, Transgenic mice

## Abstract

**Background:**

Abnormal accumulation of amyloid β-protein (Aβ) in the brain plays an important role in the pathogenesis \of Alzheimer’s disease (AD). Aβ monomers assemble into oligomers and fibrils that promote neuronal dysfunction. This assembly pathway is influenced by naturally occurring brain molecules, the Aβ chaperone proteins, which bind to Aβ and modulate its aggregation. Myelin basic protein (MBP) was previously identified as a novel Aβ chaperone protein and a potent inhibitor for Aβ fibril assembly *in vitro*.

**Methods:**

In this study, we determined whether the absence of MBP would influence Aβ pathology *in vivo* by breeding MBP knockout mice (MBP^-/-^) with Tg-5xFAD mice, a model of AD-like parenchymal Aβ pathology.

**Results:**

Through biochemical and immunohistochemical experiments, we found that bigenic Tg-5xFAD/MBP^-/-^ mice had a significant decrease of insoluble Aβ and parenchymal plaque deposition at an early age. The expression of transgene encoded human AβPP, the levels of C-terminal fragments generated during Aβ production and the intracellular Aβ were unaffected in the absence of MBP. Likewise, we did not find a significant difference in plasma Aβ or cerebrospinal fluid Aβ, suggesting these clearance routes were unaltered in bigenic Tg-5xFAD/MBP^-/-^ mice. However, MBP^-/-^ mice and bigenic Tg-5xFAD/MBP^-/-^ mice exhibited elevated reactive astrocytes and activated microglia compared with Tg-5xFAD mice. The Aβ degrading enzyme matrix metalloproteinase 9 (MMP-9), which is expressed by activated glial cells, was significantly increased in the Tg-5xFAD/MBP^-/-^ mice.

**Conclusions:**

These findings indicate that the absence of MBP decreases Aβ deposition in transgenic mice and that this consequence may result from increased glial activation and expression of MMP-9, an Aβ degrading enzyme.

## Background

One of the pathological hallmarks of Alzheimer’s disease (AD) is the abnormal accumulation amyloid β (Aβ) aggregates in brain. Aβ is a 38–43 peptide produced from the sequential proteolysis of the amyloid precursor protein (ΑβPP), a ubiquitously expressed type I membrane protein, by β secretase [1] and γ secretase [2,3]. The assembly of Aβ into soluble oligomeric forms and fibrils is proposed to have a causative role in AD through various mechanisms [4]. Soluble oligomers have been shown to correlate with synaptic plasticity and memory deficit [5,6]. Fibrillar Aβ can promote oxidative stress and neuroinflammation, and is toxic to neuronal and vascular cells [7,8].

The assembly of Aβ is influenced by a number of naturally occurring brain factors, the 'Aβ chaperone molecules’ that bind and modulate the aggregation process of the peptide. One of the better known Aβ chaperones is the apolipoprotein E (apoE) family. The apoE2 and apoE3 isoforms can suppress fibrillar Aβ deposition, while apoE4 can promote fibril formation [9,10]. Studies using transgenic mice have demonstrated that by modulating the levels of these Aβ binding partners, Aβ deposition is delayed or enhanced [11]. Other examples of Aβ chaperones include apolipoprotein J [12,13], members of heat shock proteins [14,15], α_1_-anti-chymotrypsin [16], transthyretin [17,18], proteoglycans [19], and gangliosides [20,21].

Previously, we identified myelin basic protein (MBP) as a novel Aβ chaperone that can potently inhibit its fibrillar assembly [22]. MBP is best known as a major structural protein in the central nervous system (CNS) myelin sheath. It is also suggested to have a role in intracellular signaling through interactions with membrane actin and tubulin [23]. MBPs are products of the Golli (genes of the oligodendrocyte lineage)-MBP gene complex [24]. Four major MBP isoforms are products of alternative splicing of the Golli-MBP gene complex [25,26]. The expression of the different MBP isoforms by oligodendrocytes is developmentally regulated. The predominant MBP isoform in mature human beings is 18.5 kDa [27,28]. Although the 18.5 kDa MBP undergoes post-translational modifications to give rise to eight charge isomers, its ability to bind Aβ appears to be solely sequence-dependent [22]. The strong binding of MBP to Aβ42 was demonstrated to inhibit Aβ fibril assembly in a substoichiometric molar ratio *in vitro*[29].

Interestingly, a number of studies have reported a loss of myelin and breakdown of MBP in AD patients and mouse models of AD pathology. This loss of myelin is associated with AD risk factors (for example, aging, apoE4, traumatic brain injury) [30-34] and an increase of Aβ peptides [35]. Immunolabeling of brains for Aβ showed that the most susceptible areas for its deposition are in gray matter, where little MBP is present. Conversely, areas of white matter that are abundantly supplied with MBP (for example, corpus callosum, striatum) exhibit very little Aβ deposition. Furthermore, other studies showed there was no myelin staining inside amyloid plaques [36]. Taken together, these findings suggest an inverse correlation between the levels of MBP and Aβ. However, whether MBP can actually influence Aβ accumulation *in vivo* remains unknown.

Here, we directly tested whether MBP could modulate Aβ *in vivo* by removing endogenous MBP from a mouse model of AD-like Aβ pathology. We took advantage of MBP^-/-^ mice, known as *shiverer* mice, in which no functional MBP is produced due to a gene breakage from the middle of MBP exon II [37]. MBP^-/-^ mice were crossed with human AβPP transgenic mice Tg-5xFAD, a model of parenchymal plaque amyloid pathology [38]. We show that in the absence of endogenous mouse MBP there was a significant reduction in cerebral Aβ levels and the amount of deposited fibrillar amyloid. The reduction in Aβ was not due to changes in expression or processing of human AβPP or in clearance through cerebrospinal fluid (CSF) or plasma pathways. However, in bigenic Tg-5xFAD/MBP^-/-^ mice there was a significant elevation in activated astrocytes and microglia as well as in the levels of the Aβ-degrading enzyme MMP-9. Together, these findings indicate that in the absence of MBP there is a marked reduction in Aβ pathology in Tg-5xFAD mice but that this decrease is likely to result from increased degradation via elevated neuroinflammatory glial cells and associated MMP-9.

## Methods

### Animals

All work with mice followed National Institutes of Health guidelines and was approved by the Stony Brook University Institutional Animal Care and Use Committee. Tg-5xFAD mice were obtained from Jackson Laboratories. Tg-5xFAD mice coexpress human APP and human presenilin 1 with five familial AD mutations (APP K670N/M671L + I716V + V717I and PS1 M146L + L286V) and develop early-onset Aβ accumulation and fibrillar Aβ plaques in the brain, starting at about two months of age [38]. *Shiverer* MBP^-/-^ mice were also obtained from Jackson Laboratories. *Shiverer* MBP^-/-^ mice produce no functional MBP, owing to a gene breakage from the middle of MBP exon II [37]. Hemizygous Tg-5xFAD mice were successively bred with MBP^+/-^ mice to obtain cohorts of wild-type mice, Tg-5xFAD mice, MBP^-/-^ mice, and bigenic Tg-5xFAD/MBP^-/-^ mice. 10 to 12 mice of each genotype were collected at two months of age.

### Tissue preparation

Mice were overdosed with 2.5% Avertin followed by the collection of CSF, plasma and brain. CSF was obtained following a protocol adapted from [39]. Blood was collected through heart puncture with a 27½G needle in one-tenth volume of 3.8% sodium citrate to prevent coagulation. Blood was centrifuged at 8,000*g* for 5 min at room temperature to remove platelets and cellular components. Plasma samples were stored at -80°C until analysis. Brains were perfused with PBS and bisected along the midsagittal plain. One hemisphere was snap frozen and stored at -80°C. The other hemisphere was placed in 70% ethanol, followed by xylene treatment and embedding in paraffin for immunohistochemical and histological analyses.

### ELISA analysis of cerebral Aβ peptides

The pools of Aβ_40_ and Aβ_42_ were determined by using a specific ELISA as previously described [40]. Sequential extraction of pulverized mouse forebrain tissues was as follows. To obtain a soluble fraction, tissue aliquots were homogenized with tris-buffered saline (TBS) (10 μl/mg tissue) using a bullet blender and 0.5 mm glass beads (Next Advance, Inc.) followed by 20 min centrifugation at 8,000 *g* at 4°C. The supernatant was removed as the soluble fraction and the pellet was next extracted with TBS/1% Triton X-100 following the same procedure to obtain a membrane-associated fraction. Finally, the resulting pellet was resuspended in 5M guanidine-HCl (pH 8.0), rotating at room temperature for 3 hours. After centrifugation, the supernatant was removed and kept as the insoluble fraction. Plasma was treated with 5M guanidine-HCl (pH 8.0) at room temperature for 30 min. For each fraction, a sandwich ELISA was performed, where Aβ_40_ and Aβ_42_ were captured using their respective carboxyl terminus-specific antibodies, m2G3 and m21F12, and biotinylated antibody m3D6, specific for human Aβ, was used for detection [41].

### Immunoblot analysis

The TBS/1% Triton X-100 extraction (membrane-associated fraction) was used to detect AβPP and AβPP C-terminal fragments (CTFs). Direct TBS/1% Triton X-100 extraction (total extraction) was used to detect GFAP. Protein concentration was determined using a BCA kit (Pierce). Equal amounts of total protein were separated on 4 to 12% Tris-Glycine (Invitrogen) or 16% Tricine (Invitrogen) for APP CTFs. Gels were transferred onto nitrocellulose membranes (Amersham Hybond-ECL). Membranes were blocked with 5% nonfat milk and incubated overnight at 4°C with anti-human AβPP (mouse mAb P2-1, 1:1000), anti-AβPP-CTF (rabbit pAb, 1:1,000), anti-MMP-9 (Abcam ab38898 1:1,000), anti-neurospecific β-tubulin (Abcam ab18207 1:2,000), anti-GFAP (Chemicon MAB360 1:1,000). Secondary HRP conjugated anti-mouse or anti-rabbit was used at 1:5,000 dilution. Membranes were developed using ECL (Pierce) and signals were quantified with VersaDoc (BioRad Model 3000).

### Immunohistochemical analysis

10 μm paraffin sections were deparaffinated in xylene and rehydrated with ethanol. Sections were blocked in SuperBlock blocking buffer (Thermo #37515) with 0.3% triton X-100 and incubated overnight with diluted primary antibody in 1:10 SuperBlock/PBS containing 0.1% triton X-100 at 4°C. The following antibodies were used: Aβ rabbit pAb anti-Aβ1-28 1:500), GFAP antibody (Chemicon MAB360 1:1,000), Keratan sulfate antibody (5D4) (Seikagaku Corp. 1:1,000), OC antibody (a gift from Dr. Charles Glabe, UC Irvine, 1:1,000). Antigen retrieval was done in antigen unmasking solution (Vector labs H-3301) 30 minutes at 90°C for 5D4 staining and 15 min in 88% formic acid for intraneuronal Aβ (OC) staining before blocking. Sections were treated with Alexa Fluor 488 (Invitrogen) for fluorescence staining or biotinylated secondary antibodies followed by vectastain ABC kit (Vector Labs) for DAB staining.

### Gelatin zymography

Pulverized brain aliquots were homogenized in TBS containing 1% Triton X-100 as described. After centrifugation, 400 μl of supernatant was incubated with 50 μl 50% pre-washed gelatin agarose beads and allowed to rotate overnight at 4°C. The beads were then pelleted by centrifugation. After removing the supernatant, the beads were washed in PBS and eluted with 50 μl 1X gel loading dye. Half of the elution was separated on 7.5% SDS-PAGE containing 0.1% gelatin. Following electrophoresis, the gels were gently agitated in 2.5% Triton X-100 at room temperature. The buffer was changed every 40 min for three times. After briefly rinsed in the assay buffer, gels were incubated with shaking in assay buffer (50 mM Tris, 0.2M NaCl, 6.7mM CaCl_2_) at 37°C for 20 hours. Gels were stained with Coomassie blue and destained until clearing by gelatinases was visible.

### Statistical analysis

Data were analyzed using the unpaired two-tailed Student’s *t* test. Error bars represent standard error of the mean (SEM). Significance was taken when *P* value was less than 0.05.

## Results

### Significant reduction of insoluble cerebral Aβ in Tg-5xFAD/MBP^-/-^ mice

**Figure 1:**
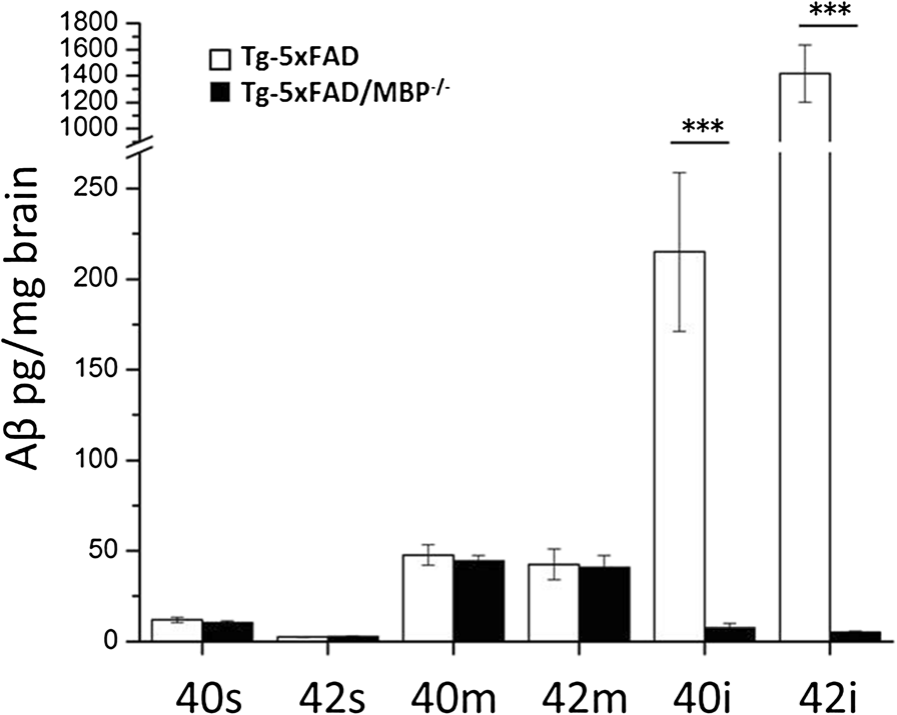
**Aβ ELISA of Tg-5xFAD and Tg-5xFAD/MBP**^**-/- **^**mice of different extraction pools.** Pulverized brain was sequentially extracted with TBS buffer, TBS with 1% Triton X-100, and 5M Guanidine-HCl for soluble, membrane, and insoluble Aβ. The amount of Aβ did not differ in soluble and membrane fractions but was significantly decreased in the insoluble fraction of bigenic Tg-5xFAD/MBP^-/-^ mice. The reduction was ≈ 8-fold for Aβ40 and ≈ 30-fold for Aβ42. Data presented are the mean ± SEM. of 10 or 11 mice per group. *** *P* = 0.00013. i, insoluble; m, membrane-associated; s, soluble.

Previously, we identified MBP as a potent Aβ fibrillogenesis inhibitor *in vitro* via a sequence-dependent interaction [22,29,42]. To investigate whether the absence of MBP could influence Aβ pathology *in vivo*, we bred MBP^-/-^ mice to Tg-5xFAD mice, a model of parenchymal AD-like Aβ pathology. Age-matched Tg-5xFAD and Tg-5xFAD/MBP^-/-^ mice were collected at 2 months of age when thioflavin S-positive fibrillar plaques begin to appear in Tg-5xFAD mice [43]. Pulverized brain aliquots were sequentially extracted into soluble (s), membrane-associated (m) and insoluble (i) fractions for Aβ ELISA analysis. Bigenic Tg-5xFAD/MBP^-/-^ mice had a significant reduction in the amount of insoluble Aβ, with an eight-fold reduction in Aβ_40_ and a 30-fold reduction in Aβ_42._ No significant differences were found in the levels of soluble Aβ and membrane-associated Aβ between Tg-5xFAD and Tg-5xFAD/MBP^-/-^ mice (Figure 1).

### parenchymal Aβ deposition in Tg-5xFAD/MBP^-/-^ mice

**Figure 2:**
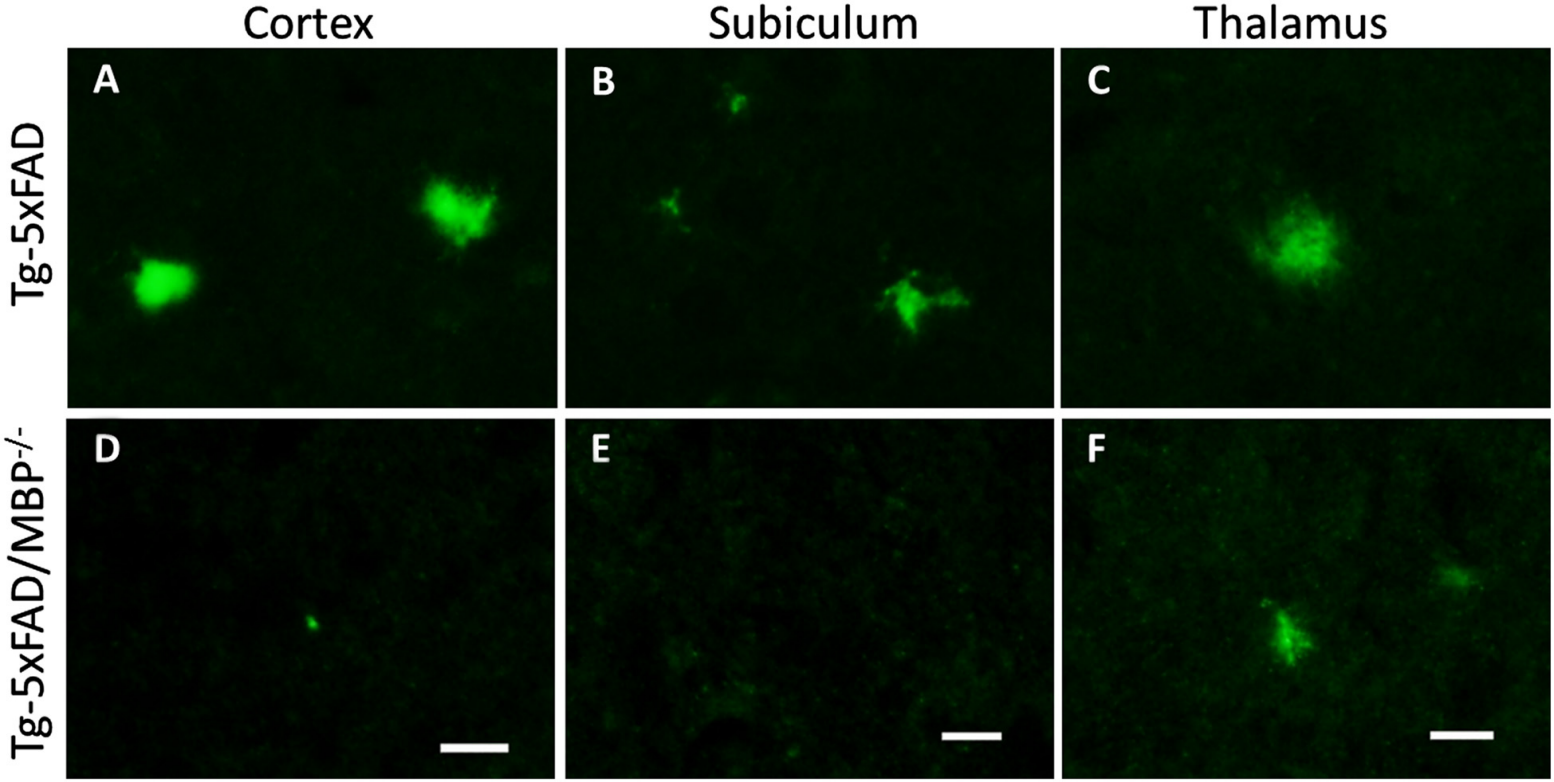
**Immunolabeling of brain Aβ deposits in young Tg-5xFAD and bigenic Tg-5xFAD/MBP**^**-/- **^**mice.** Rabbit polyclonal antibody against Aβ_1-28_ was used to detect Aβ deposits. Bigenic Tg-5xFAD/MBP^-/-^ mice (bottom panels) had a marked decrease in Aβ deposition compared to Tg-5xFAD mice (top panels) in: cortex **(A, D)**, subiculum **(B, E)** and thalamus **(C, F)**. Scale bars, 10 μm.

We next performed immunofluorescent labeling for Aβ on brain sections from Tg-5xFAD and bigenic Tg-5xFAD/MBP^-/-^ mice using an anti-Aβ N-terminal antibody. Even though Aβ deposition just begins at this early age in Tg-5xFAD mice, there was a remarkable decrease in both number and the size of Aβ plaques in bigenic Tg-5xFAD/MBP^-/-^ mice observed in the cortex, subiculum, and thalamus (Figure 2), which was consistent with the reduction of insoluble Aβ from the ELISA analysis.

### The absence of MBP does not alter human AβPP protein levels or processing by α and β secretases in Tg-5xFAD mice

**Figure 3:**
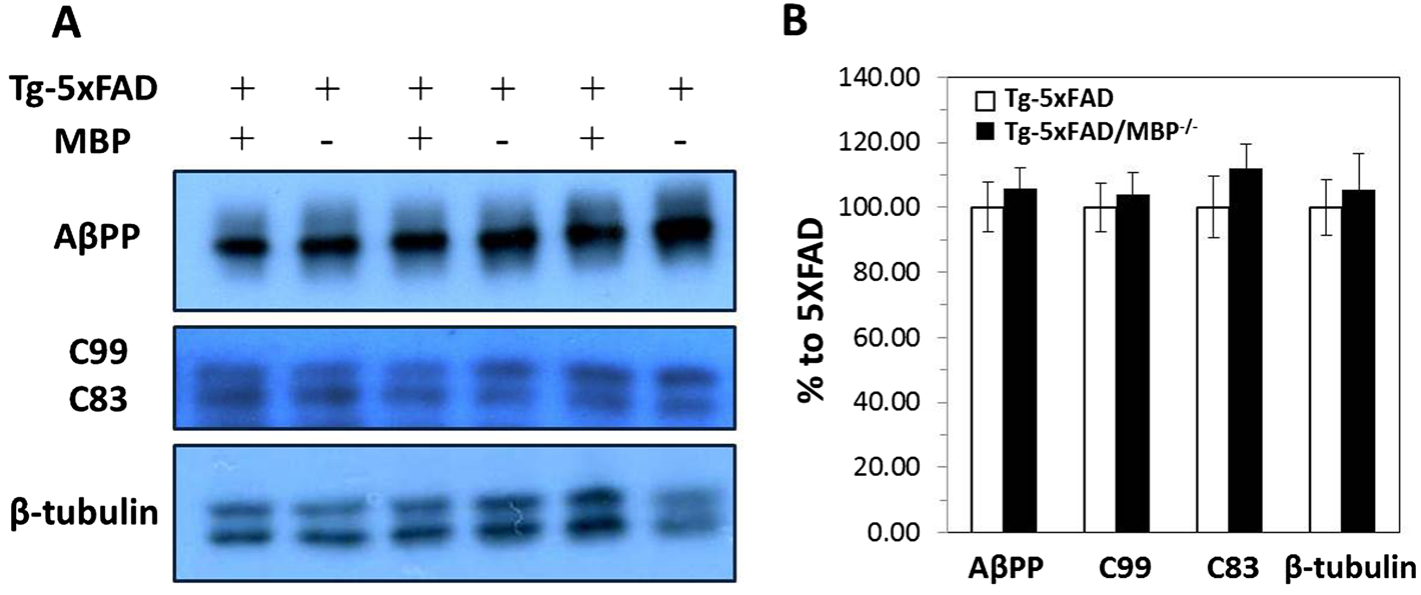
**Absence of MBP does not alter AβPP expression or processing in Tg-5xFAD/MBP**^**-/- **^**mice. (A)** Equal amount of total protein was separated on 4 to 12% or Tris-Glycine gel for AβPP or 16% Tricine for AβPP CTFs. **(B)** The chemiluminescence signals were quantified and presented as percentage of Tg-5xFAD. Data presented are the mean ± SEM of 11 or 12 mice per group.

The observed reduction in Aβ levels and deposition could be a consequence of decreased Aβ production or increased Aβ catabolism. To determine whether the absence of MBP led to a decrease in human AβPP expression or processing in Tg-5xFAD mice, we performed quantitative immunoblotting on membrane-associated fractions using antibodies against human AβPP and the AβPP CTFs generated from α secretase (C83) and β secretase cleavages (C99) (Figure 3A). There were no significant differences in the levels of human AβPP, or in the levels of AβPP CTF cleavage products, between Tg-5xFAD and Tg-5xFAD/MBP^-/-^ mice (Figure 3B). This finding suggests that the reduction of Aβ in bigenic Tg-5xFAD/MBP^-/-^ mice was unlikely to have resulted from decreased Aβ production.

### The levels of intracellular Aβ are unaltered between Tg-5xFAD and Tg-5xFAD/MBP^-/-^mice

**Figure 4:**
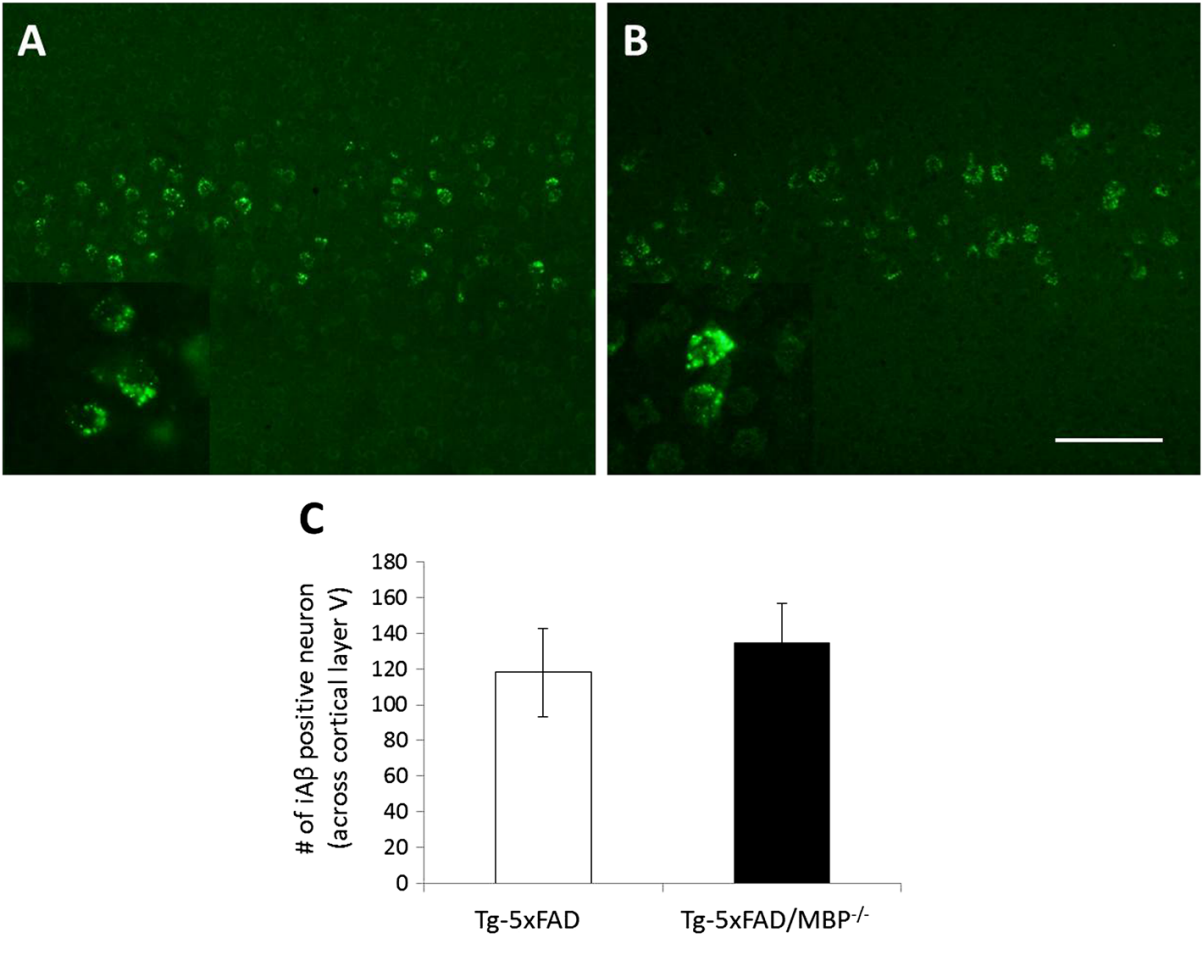
**No significant difference in the level of intraneuronal Aβ between Tg-5xFAD and Tg-5xFAD/MBP**^**-/- **^**mice.** To assess the level of intraneuronal Aβ, sections were pretreated with formic acid and incubated with an oligomer specific antibody, OC. Prominent cell body Aβ staining was observed in layer V of cortex in both **(A)** Tg-5xFAD and **(B)** Tg-5xFAD/MBP^-/-^ mice. **(C)** Cortical neurons with positive iAβ were counted, no difference was observed between different genotypes. Scale bar, 100 μm. Data presented are the mean ± SEM of 4 or 5 mice per group.

We next evaluated the level of intracellular Aβ (iAβ), since its accumulation is proposed to precede extracellular Aβ deposition and it is suggested as one of the first events in the progression of Aβ pathology [44,45]. The detection of iAβ has been controversial, owing to the cross-reaction of some Aβ antibodies with AβPP. To avoid this potential confound, we used a conformational antibody (OC), which is specific to a fibrillar epitope present in Aβ oligomers and fibrils [46]. We saw prominent iAβ-containing neurons in the cortical layer V that appeared comparable between Tg-5xFAD and Tg-5xFAD/MBP^-/-^ mice (Figures 4A,B). The numbers of cortical neurons that were positive with iAβ were counted (Figure 4C). At the age of two months, male mice had three-fold less iAβ positive neurons than female mice, but there was no significant difference between Tg-5xFAD and Tg-5xFAD/MBP^-/-^ mice of the same sex. This result and the quantitative data from Figure 3 together indicate that the absence of MBP does not alter Aβ production and suggest that the events causing Aβ reduction in Tg-5xFAD/MBP^-/-^ mice probably occur extracellularly, after Aβ is released.

### Efflux of Aβ into plasma or CSF is unaltered in Tg-5xFAD/MBP^-/-^ mice

**Figure 5:**
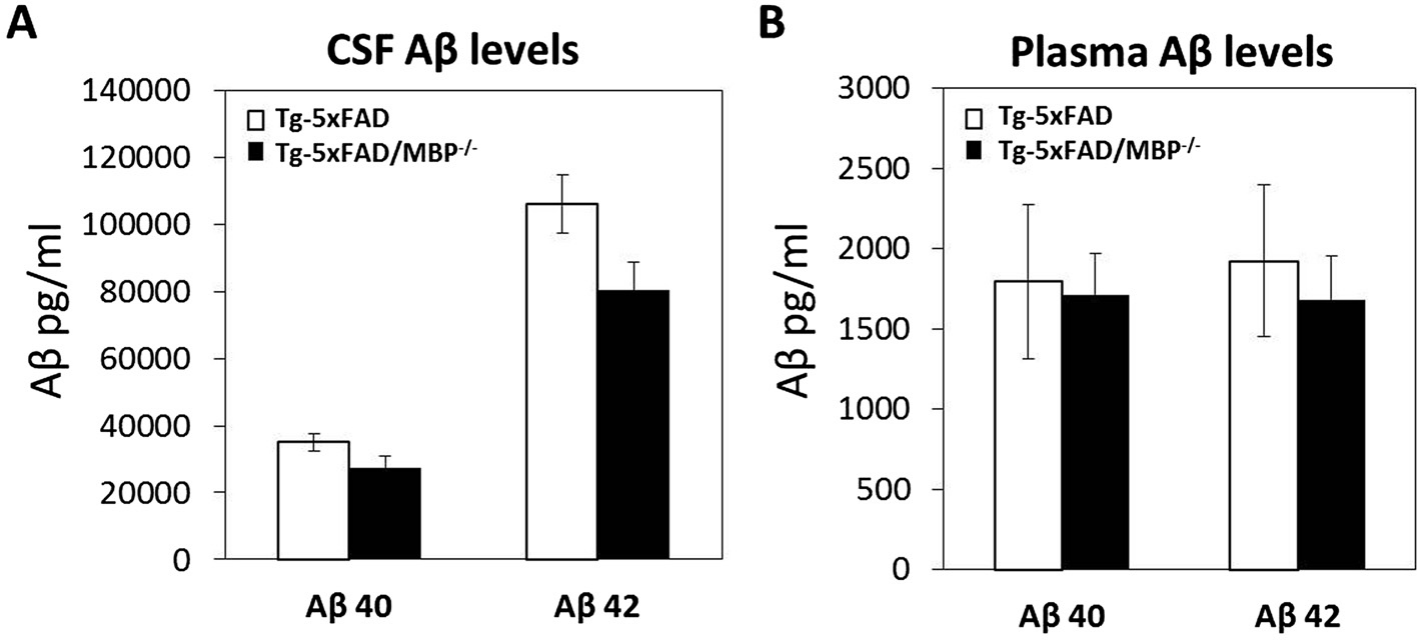
**No significant difference in plasma and CSF Aβ levels of Tg-5xFAD and Tg-5xFAD/MBP**^**-/- **^**mice.** ELISA measurements were performed for Aβ40 and Aβ42 in plasma samples **(A)** and CSF samples **(B)** collected from Tg-5xFAD and Tg-5xFAD/MBP^-/-^ mice. The data presented are the mean ± SEM of ten mice per group.

Efflux into plasma or CSF represents major clearance pathways for Aβ in brain [47-49]. To determine whether the efflux of Aβ was affected by the absence of MBP, we performed ELISA analyses for Aβ on guanidine-extracted plasma samples and CSF samples obtained from the two groups of mice. There were similar levels of Aβ40 and Aβ42 in the plasma of Tg-5xFAD and Tg-5xFAD/MBP^-/-^ mice (Figure 5A). Likewise, in CSF there was no significant difference in the levels of Aβ40 and Aβ42 (Figure 5B). These findings suggest that there is no enhancement of plasma or CSF clearance of Aβ in the absence of MBP.

### Elevated neuroinflammatory cells in Tg-5xFAD/MBP^-/-^ mice

**Figure 6:**
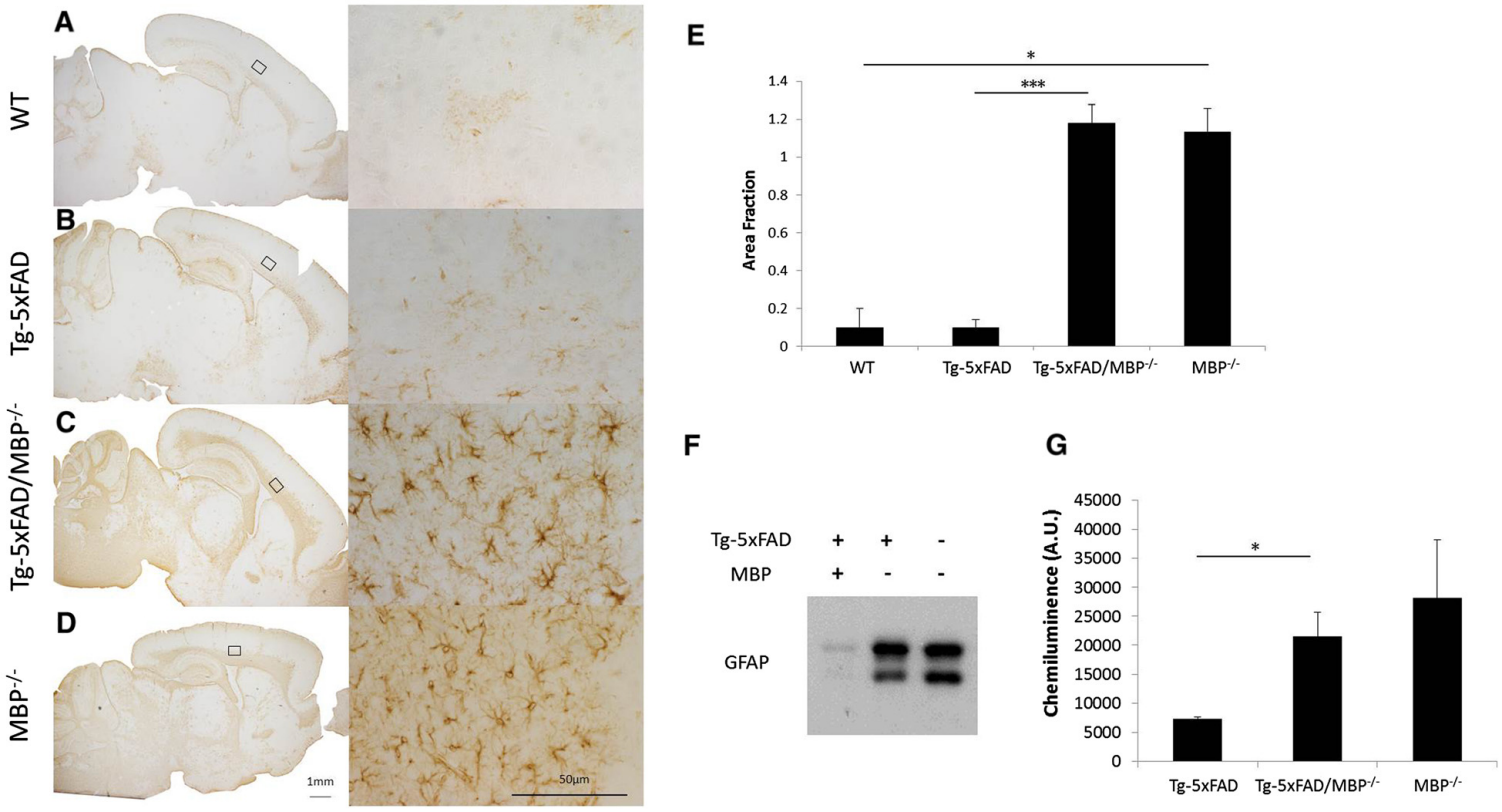
**Increased reactive astrocytes in Tg-5xFAD/MBP**^**-/- **^**mice are associated with the absence of MBP.** Immunostaining for GFAP showed increased reactive astrocytes in Tg-5xFAD/MBP^-/-^ mice **(C)** compared with wild-type mice **(A)** and Tg-5xFAD mice **(B)**. Increased reactive astrocytes were also observed in MBP^-/-^ mice **(D)**. Left panels, overviews (scale bar, 1 mm); right panels, higher magnification (scale bar, 50 μm). **(E)** Levels of immunostaining of the selected areas in **(A)** to **(D)** were quantified by Image J software and represented as % of area. Data presented are the mean ± SEM of 3 to 5 mice per group. *P = 0.014, ***P = 0.00,003. **(F)** Representative immunoblots for GFAP in total brain homogenates from the different mice. **(G)** Quantitation of GFAP from immunoblots. Data presented are the mean ± SEM of three mice per group. *P = 0.03.

Activated glial cells are known to participate in Aβ clearance by producing a number of Aβ degrading enzymes. Immunostaining for astrocytes using an antibody to GFAP, we found that bigenic Tg-5xFAD/MBP^-/-^ mice (Figure 6C) had extensive astrocyte staining compared with wild-type mice (Figure 6A) and Tg-5xFAD mice (Figure 6B). In addition, we observed a similar elevated astrocyte immunostaining pattern in the MBP^-/-^ mice (Figure 6D) compared with wild-type mice, as reported previously [50,51]. The elevated GFAP was also confirmed by quantitative immunoblotting on brain homogenates from the different mice (Figure 6E). GFAP levels were increased three- to four-fold in Tg-5xFAD/MBP^-/-^ mice and MBP^-/-^ mice compared with Tg-5xFAD mice (Figure 6F).

**Figure 7:**
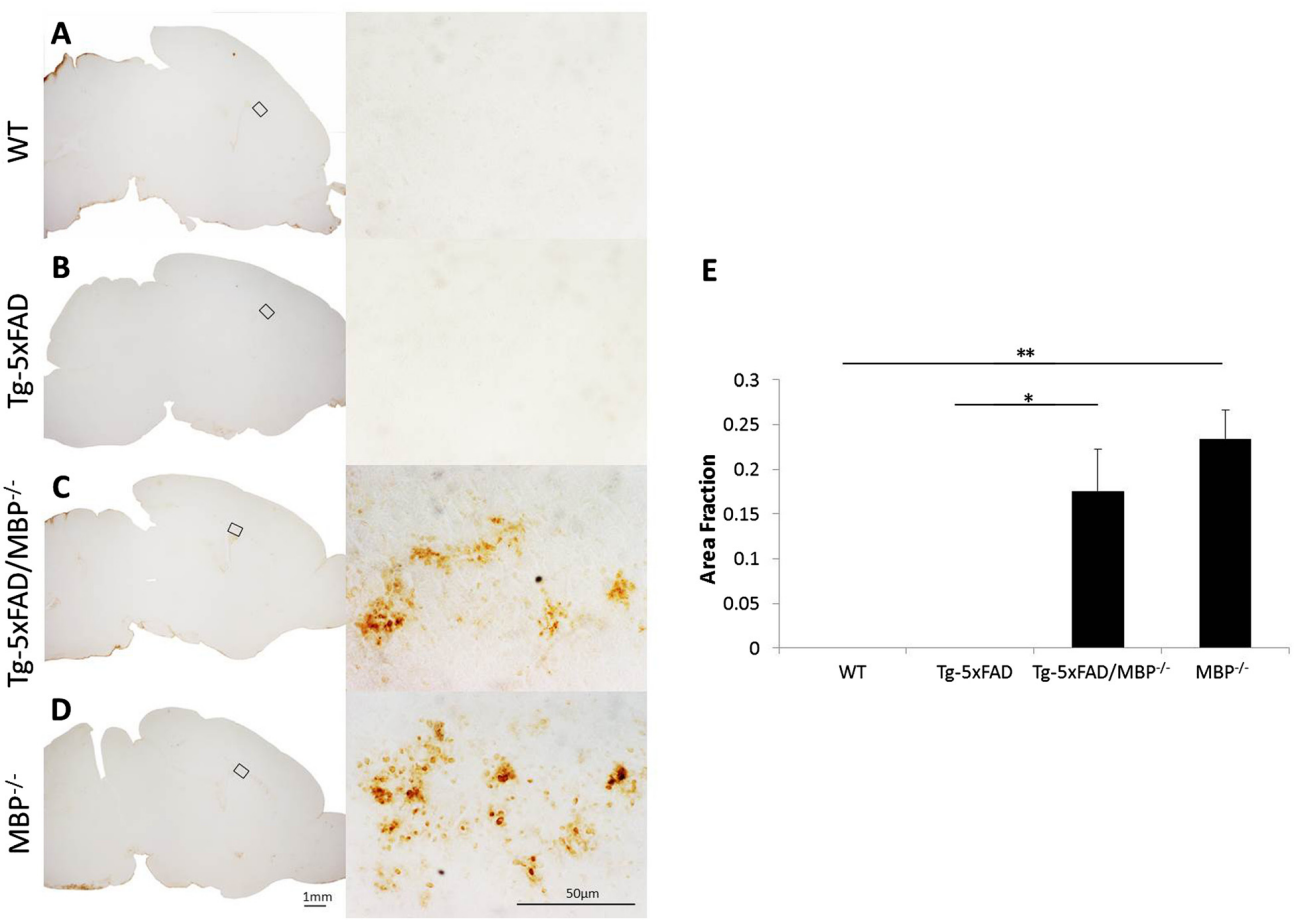
**Increased activated microglia in Tg-5xFAD/MBP**^**-/- **^**mice are associated with the absence of MBP.** Immunostaining with 5D4 keratan sulfate antibody showed increased activated microglia in Tg-5xFAD/MBP^-/-^ mice **(C)** compared with wild-type mice **(A)** and Tg-5xFAD mice **(B)**. Increased activated microglia were also observed in MBP^-/-^ mice **(D)**. Left panels, overviews (scale bar, 1 mm); right panels, higher magnification (scale bar, 50 μm). **(E)** Levels of immunostaining of the selected area in **(**A**)** to **(**D**)** were quantified by Image J software and represented as % area fraction. Data presented are the mean ± SEM of 3 to 5 mice per group. *P = 0.0106, **P = 0.0123.

Similarly, using a marker for activated microglia showed increased immunostaining in bigenic Tg-5xFAD/MBP^-/-^ mice (Figure 7C) compared with wild-type mice (Figure 7A) and Tg-5xFAD mice (Figure 7B). Again, we observed a similar elevated activated microglial immunostaining pattern in MBP^-/-^ mice (Figure 7D). Together, these findings indicate that bigenic Tg-5xFAD/MBP^-/-^ mice have elevated levels of neuroinflammatory cells compared with Tg-5xFAD mice and that this effect appears to be associated with the absence of MBP, as MBP^-/-^ mice exhibit comparable increases.

### Increased expression of the Aβ degrading enzyme MMP-9 in Tg-5xFAD/MBP^-/-^ mice

**Figure 8:**
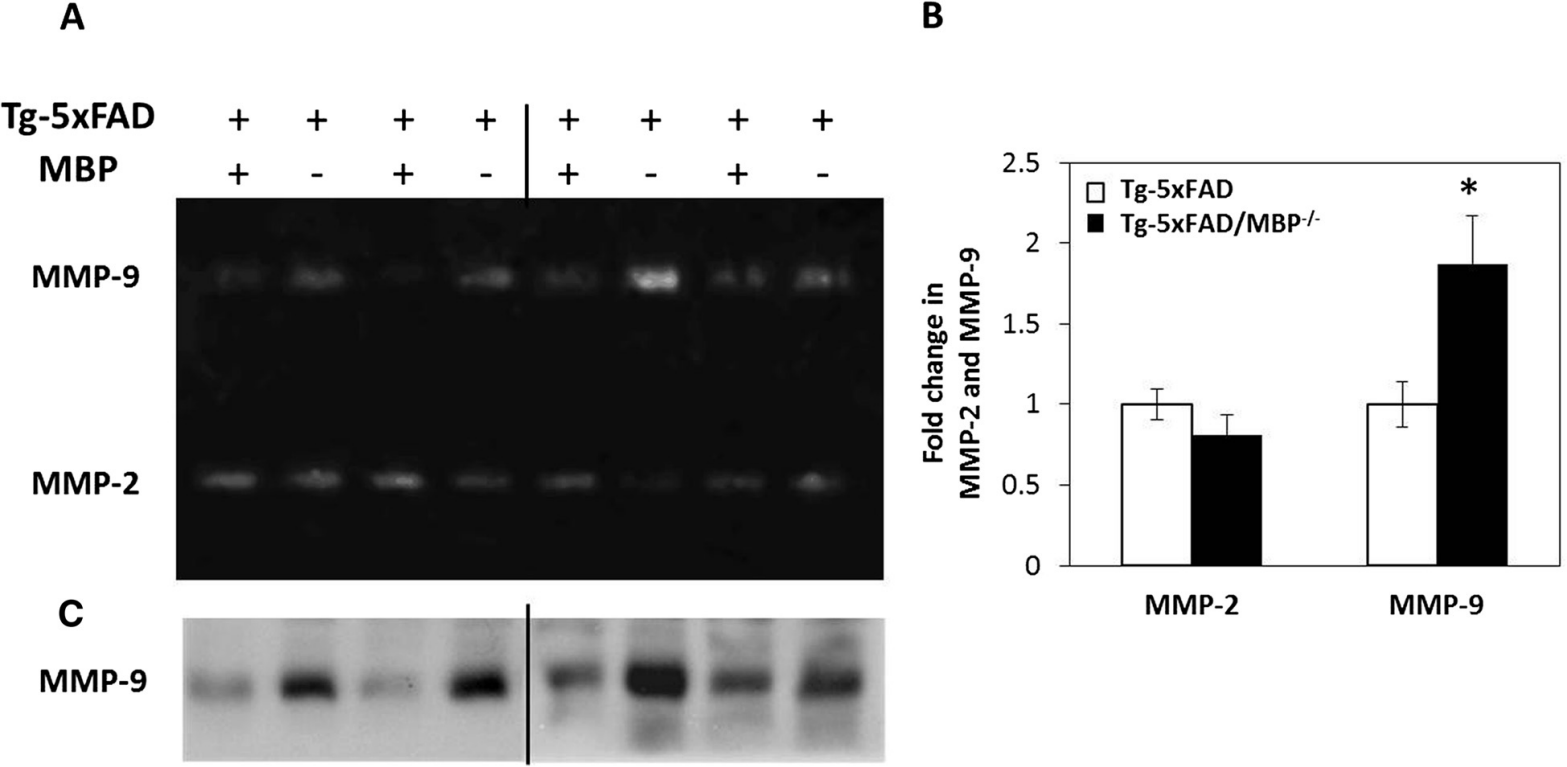
**Elevated MMP-9 levels in Tg-5xFAD/MBP**^**-/- **^**mice. (A)** Gelatin zymography of brain homogenates shows increased levels of MMP-9, but not MMP-2, in bigenic Tg-5xFAD/MBP^-/-^ mice. **(B)** Quantification of gelatin zymography signals show MMP-9, but not MMP-2, was increased 2-fold in bigenic Tg-5xFAD-MBP^-/-^ mice. Data presented are mean ± SEM of four mice per group. *p < 0.05. **(C)** Immunoblot of MMP-9 in brain homogenates of Tg-5xFAD mice and bigenic Tg-5xFAD/MBP^-/-^ mice.

Reactive astrocytes and activated microglia are known to express the Aβ-degrading enzymes matrix metalloproteinase 2 and 9 (MMP-2 and MMP-9) [52-54]. To measure the activity of MMP-2 and MMP-9, gelatin zymography was performed using brain lysates prepared from Tg-5xFAD mice and bigenic Tg-5xFAD/MBP^-/-^ mice that were concentrated by filtration over gelatin agarose. Gelatin zymography showed a two-fold increase in MMP-9, but not MMP-2, in Tg-5xFAD/MBP^-/-^ mice compared with Tg-5xFAD mice (Figure 8A,B). Immunoblotting showed a similar increase in MMP-9 protein levels in Tg-5xFAD/MBP^-/-^ mice (Figure 8C).

## Discussion

The assembly pathway of endogenous Aβ is influenced by various Aβ chaperone proteins, which can either promote or inhibit the aggregation of Aβ. Previously, our *in vitro* work showed that MBP could strongly bind fibrillogenic forms of Aβ and potently inhibit their assembly into fibrils [22,29]. Although MBP is largely embedded in myelin sheaths, it can be readily detected in the CSF of healthy individuals within the range of μg/l [55,56]. Furthermore, after brain injuries and myelin breakdown, the MBP is increased in the CSF [55,57,58]. Golli-MBP proteins are expressed in several cell types in the CNS, including oligodendrocytes, neurons, and microglia [59-61]. Recent studies have implicated Golli-MBP proteins as multifunctional intracellular scaffolds that can bind a number of intracellular proteins and small molecule ligands affecting diverse cellular processes [62]. These findings suggest that cells that express Golli-MBP proteins could affect intracellular, and possibly extracellular, Aβ assembly, molecular interactions, and associated pathogenic effects. Thus with its abundance in the brain and its close proximity to Aβ it is possible that MBP could influence Aβ levels, especially at early stages of myelin breakdown.

In this present study, we sought to explore the consequences of removing endogenous MBP on Aβ accumulation in transgenic mice. To do this, we generated Tg-5xFAD/MBP^-/-^ mice by breeding MBP^-/-^ mice to Tg-5xFAD mice, a model of early-onset parenchymal Aβ pathology. Owing to the short life span of MBP^-/-^ mice [63], the choice of an aggressive Aβ depositing mouse model such as Tg-5xFAD was necessary to allow us to investigate Aβ pathology at a young age. At weaning age, the bigenic Tg-5xFAD/MBP^-/-^ mice exhibited the same severe shivering phenotype that is characteristic of the MBP^-/-^ mice, but died at a younger age before reaching three months, which we suspected to be a result of the rapid Aβ accumulation from the FAD mutations.

Based on our earlier *in vitro* data, showing a potent inhibitory effect of MBP on Aβ fibril assembly, we might have expected to see an increase in Aβ accumulation and deposition in the absence of MBP. Conversely, in the absence of MBP the Tg-5xFAD mice exhibited significantly decreased Aβ levels and Aβ deposition in the brain at two months of age (Figure 1 and Figure 2, respectively). However, this finding was not unique to Tg-5xFAD/MBP^-/-^ mice. We bred Tg-SwDI mice, another model of early-onset Aβ accumulation and deposition, with MBP^-/-^ mice. Like bigenic Tg-5xFAD/MBP^-/-^ mice, bigenic Tg-SwDI/MBP^-/-^ mice also exhibited decreased Aβ levels and Aβ deposition at 2 to 3 months of age (data not shown).

A lowering of cerebral Aβ levels can result from reduced expression of AβPP and production of the peptide. However, the levels of AβPP protein, AβPP CTFs and the presence of intraneuronal Aβ were similar in the brains of Tg-5xFAD mice and bigenic Tg-5xFAD/MBP^-/-^ mice, suggesting that the reductions in Aβ were not the consequence of decreased production in the absence of MBP. Alternatively, Aβ reductions in the brain can arise, owing to increased clearance through established efflux pathways. For example, through one route Aβ initially released by neurons enters the interstitial fluid, which drains to the CSF [48]. Yet another mechanism involves active transport of Aβ across the blood–brain barrier into the circulation that is mediated by known Aβ receptors, including low-density lipoprotein receptor-related protein 1 (LRP1) and P-glycoprotein [64,65]. However, we found no increase in the levels of Aβ in the CSF or plasma of bigenic Tg-5xFAD/MBP^-/-^ mice, arguing against increased Aβ efflux in the absence of MBP.

Another recognized clearance mechanism of Aβ in brain involves a broad class of Aβ-degrading enzymes that are largely released by activated neuroinflammatory cells [66]. In the bigenic Tg-5xFAD/MBP^-/-^ mice, we found markedly elevated staining for reactive astrocytes and activated microglia compared with Tg-5xFAD mice (Panels C of Figures 6 and 7). Once Tg-5xFAD mice age and develop numerous fibrillar Aβ plaques, they exhibit a robust neuroinflammatory response to these plaques that is characterized by reactive astrocytes and activated microglia [38]. Yet, in our study the young animals at two months of age are just beginning to develop amyloid plaques and neuroinflammatory cells are scarce or absent (Panels B of Figures 6 and 7). Furthermore, the increased immunostaining of activated glial markers in bigenic Tg-5xFAD/MBP^-/-^ mice was not observed around the few plaques that had developed. However, the increase in neuroinflammatory cells was similarly observed in the MBP^-/-^ mice alone (Panels D of Figures 6 and 7); this is consistent with previous reports regarding these mice [51]. Activation of glial cells is a common phenomenon in neurodegenerative diseases, including AD, multiple sclerosis, and traumatic brain injury. A number of other mouse models with deficiencies in myelination from different causes including *jimpy, MBP*^*mld*^, and *quaking* were all found to exhibit glial activation [67,68]. Although it is not well understood, the increase in neuroinflammatory cells observed in the MBP^-/-^ mice appears to be a pleiotropic effect due to the loss of MBP and abnormal myelination, thereby disrupting normal interaction between glial cells. Indeed, it has been suggested that a proliferation of mixed-phenotype glial cells, which were found to be increased in the pathogenic white matter, contribute to this gliosis in MBP^-/-^ mice [69].

In any case, reactive astrocytes and activated microglia both produce MMP-2 and MMP-9 in a number of CNS disorders, including AD and multiple sclerosis [70-73]. In regards to Aβ-degrading enzymes, MMP-2 and MMP-9 are distinctive in that both can degrade soluble Aβ peptides and fibrillar plaque Aβ [74-78]. While we did not find elevated MMP-2 levels in the brains of bigenic Tg-5xFAD/MBP^-/-^ mice, we did observe a significant increase in MMP-9 levels, as assessed by zymography and immunoblotting (Figure 8). Although it is possible that other Aβ-degrading enzymes could be elevated in Tg-5xFAD/MBP^-/-^ mice, quantitative PCR analysis did not reveal increased expression of some of the more common enzymes including insulin-degrading enzyme, neprilysin, or angiotensin-converting enzyme (data not shown). This suggests that elevated MMP-9, produced by reactive astrocytes and activated microglia as a consequence of the absence of MBP, could contribute to the decreased Aβ levels observed in Tg-5xFAD/MBP^-/-^ mice.

A goal of this study was to investigate the potential consequences of MBP-Aβ interactions in the brain. Based on our previous *in vitro* work showing that MBP strongly binds Aβ and inhibits fibrillar assembly [22,29,42], one prediction is that in the absence of MBP there could be greater accumulation of fibrillar Aβ. On the contrary, as shown here, in the complete absence of MBP there was a significant reduction in the accumulation of Aβ. However, the Aβ reduction observed in the bigenic Tg-5xFAD/MBP^-/-^ mice is more likely to be an indirect pleiotropic effect of the absence of MBP and proper myelination, leading to glial activation and increased Aβ degrading enzymes, rather than a consequence of the direct loss of interaction between MBP and Aβ. Recently, we identified specific residues in MBP that are essential for Aβ binding and fibril assembly inhibition [79]. To overcome the significant limitations of MBP^-/-^ mice, future efforts are focused on utilizing novel mice that harbor specific mutations of the residues in MBP involved in Aβ binding and fibril inhibition. In contrast with the MBP^-/-^ mice, these new mutant MBP mice are largely expected to retain normal physiological functions of MBP but will be devoid of the ability to interact with Aβ. Alternatively, efforts are also focused on characterizing novel transgenic mice that over-express biologically active fragment of MBP in brain. Together, the novel MBP knock-in mutant mice and MBP-expressing transgenic mice, crossed with AβPP transgenic mice, will provide more suitable models for studying *in vivo* MBP-Aβ relationships, thereby enabling us to gain insight into Aβ assembly in brain and a potential therapeutic role of MBP as an Aβ fibril assembly inhibitor.

## Conclusions

The primary findings of this study show that in the absence of MBP there is decreased accumulation and deposition of Aβ in Tg-5xFAD mice. The decrease in Aβ was not a consequence of reduced AβPP expression or processing or of increased peptide clearance through plasma and CSF efflux pathways. However, there were elevated reactive astrocytes and microglia accompanied by increased expression of Aβ-degrading enzyme MMP-9 in bigenic Tg-5xFAD/MBP^-/-^ mice. The absence of MBP promotes a neuroinflammatory environment that can reduce Aβ accumulation in the brains of transgenic mice.

## Abbreviations

AβPP: amyloid precursor protein; Aβ: amyloid beta protein; apoE: apolipoprotein E; MBP: myelin basic protein; C83: C-terminal fragment of AβPP generated by α secretase cleavage; C99: C-terminal fragment of AβPP generated by β secretase cleavage; CNS: central nervous system; CSF: cerebrospinal fluid; CTF: C-terminal fragment; ELISA: enzyme-linked immunosorbent assay; GFAP: glial fibrillary acidic protein; iAβ: intracellular amyloid beta protein; MBP: myelin basic protein; MMP: matrix metalloproteinase; OC: a fibril specific, conformation dependent antibody used in intracellular Aβ detection; PBS: phosphate-buffered saline; SEM: standard error of the mean; TBS: tris-buffered saline.

## Competing interests

Both authors declare that they have no competing interests.

## Authors’ contributions

MO designed, performed experiments and analyzed the data. WEVN obtained funding, helped conceive the project and interpreted data. Both authors read and approved the final manuscript.
